# *Limnofasciculus delicatus* (Coleofasciculaceae, Coleofasciculales), a Novel Mat‐Forming Cyanobacterium From Shenandoah River, Virginia, USA

**DOI:** 10.1111/1758-2229.70377

**Published:** 2026-06-21

**Authors:** Rosalina Stancheva, Cecilio Valadez‐Cano, Benoit Van Aken, Gordon M. Selckmann, Janice Lawrence, A. Bruce Cahoon

**Affiliations:** ^1^ Department of Environmental Science and Policy Potomac Environmental Research and Education Center, George Mason University Fairfax and Woodbridge Virginia USA; ^2^ Department of Biology University of New Brunswick Fredericton New Brunswick Canada; ^3^ Department of Chemistry and Biochemistry Potomac Environmental Research and Education Center, George Mason University Fairfax and Woodbridge Virginia USA; ^4^ Interstate Commission on the Potomac River Basin Rockville Maryland USA; ^5^ Department of Natural Sciences The University of Virginia's College at Wise Wise Virginia USA

**Keywords:** benthic cyanobacterial proliferations, freshwater flowing systems, metagenome‐assembled genomes, phylogenomics

## Abstract

Benthic cyanobacterial mats in flowing waters are complex communities typically composed of taxa from the orders Coleofasciculales and Oscillatoriales, many of which have unresolved taxonomic positions and poorly characterized toxic potential. We collected field mats of a benthic non‐heterocytous filamentous cyanobacterium from the North and South Forks of the Shenandoah River in Northern Virginia (USA) that were dominated by a novel morphotype. Whole‐genome and 16S rRNA gene phylogenetic analyses placed this cyanobacterium within the recently described genus *Limnofasciculus* (Coleofasciculaceae). Genome‐based species delimitation metrics fell below accepted thresholds for bacterial species delineation relative to *Limnofasciculus baicalensis*, the only formally described species in the genus to date, supporting recognition of the cyanobacterium from Shenandoah River as a distinct species. Furthermore, the two *Limnofasciculus* species exhibited marked structural differences in the Box B helix of the 16S–23S ITS region. Comparative genomic analyses revealed a genome size similar to *L. baicalensis* and a conserved core gene repertoire alongside substantial divergence in biosynthetic gene cluster composition. Neither species contains biosynthetic gene clusters associated with the production of known cyanotoxins. Based on morphological, phylogenetic and genomic evidence, we describe *Limnofasciculus delicatus* sp. nov., supported by light microscopy and whole‐genome characterization.

## Graphical abstract

A novel mat‐forming benthic freshwater cyanobacterium, *Limnofasciculus delicatus*, is described based on morphological, phylogenetic and genomic evidence from the North and South Forks of the Shenandoah River. Although *Limnofasciculus* *delicatus* lacks biosynthetic gene clusters associated with the production of known cyanotoxins, it occurs in association with anatoxin‐producing benthic cyanobacteria.

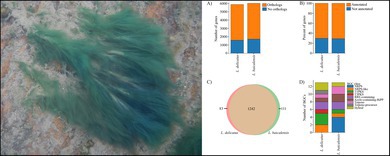

## 1 Introduction

Benthic cyanobacterial mat proliferations in flowing waters are increasing worldwide, threatening ecosystem stability, wildlife and domestic animals due to their high biomass and toxin production (Wood et al. 2020). In streams, the most abundant benthic neurotoxin producers include species of *Microcoleus*, the primary source of anatoxins, which have been linked to domestic animal mortality and adverse impacts on aquatic life (Kelly et al. 2026; Valadez‐Cano et al. 2023). This genus is common in the Shenandoah River (Northern Virginia, USA), which over the past decade has experienced increasing blooms of nuisance filamentous green algae and harmful benthic cyanobacteria, posing risks to recreational use, aquatic ecosystems and raw water supplies (Buchanan et al. 2021). The Shenandoah River, located in the Mid‐Atlantic region of the United States (Virginia and West Virginia), is the largest tributary of the Potomac River. Detection of toxins (anatoxin‐a and microcystins at concentrations below advisory thresholds) led to river closures in 2021 and 2022 and prompted a subsequent pilot investigation of benthic cyanobacterial taxonomic composition (Virginia Department of Environmental Quality 2021, 2023, 2026).

*Microcoleus*‐dominated mats are associated with diverse benthic assemblages sometimes comprising multiple cyanobacterial taxa, including members of the family Microcoleaceae and consistently include representatives of the family Coleofasciculaceae, which have been repeatedly detected through molecular surveys (Valadez‐Cano, Tromas, et al. 2025; Sohrab, personal communication).

The family Coleofasciculaceae (Komárek et al. 2014), with the type genus *Coleofasciculus* (Siegesmund et al. 2008), includes taxa characterized by filament bundles enclosed within a common sheath and trichomes with apical cells lacking calyptra. However, the recent expansion of this family has highlighted the need for taxonomic revision and refinement (Strunecký et al. 2023). Members of Coleofasciculaceae are predominantly found in terrestrial, wetland and marine intertidal environments, although several new freshwater genera have been described in recent years based on molecular evidence (Fernandes et al. 2021; Radzi et al. 2021; Jusko et al. 2024; Wu et al. 2024). The recently described genus *Limnofasciculus* from Lake Baikal has further expanded the ecological breadth of this family (Sorokovikova et al. 2023). To date, there is no evidence that members of Coleofasciculaceae produce the major cyanotoxins commonly monitored for water quality (e.g., microcystins, anatoxin‐a, cylindrospermopsin or saxitoxins), although biosynthetic gene clusters (BGCs) potentially encoding other secondary metabolites have been reported in *Limnofasciculus* (Sorokovikova et al. 2023).

Preliminary observations indicate that one of the most abundant cyanobacteria covering large areas of the Shenandoah River bottom consists of filamentous forms morphologically consistent with members of Coleofasciculaceae. However, due to the taxonomic ambiguity within this family, particularly for taxa co‐occurring with anatoxin‐producing *Microcoleus*, its identity and ecological role remain unclear.

The aims of this study were to: (1) determine the taxonomic placement of this abundant cyanobacterial morphospecies from the Shenandoah River using molecular phylogenetic analyses and comparative genomics; (2) assess the presence of genes and pathways associated with toxin production, and (3) formally describe the species, including its field characteristics and microscopic morphology, to facilitate consistent identification.

## 2 Materials and Methods

### 2.1 Sampling Sites and Cyanobacteria Collection

The Shenandoah River has two forks (South and North), each approximately 160 km long, which merge into the 89.5 km long Shenandoah River (US Geological Survey 2025). We obtained individual fresh mats of filamentous non‐heterocytous cyanobacteria from two river locations in 2024 and 2025. We used two field mat samples for this study as follows. Sample 1 was collected from the North Fork Shenandoah River near Strasburg (site NF11 with coordinates 38.97314, −78.35188) on 12 June 2024 by Gordon M. Selckmann. Sample 2 was collected from the South Fork Shenandoah River upstream of Front Royal (38.86766, −78.28067) on 13 August 2025 by Jacob Mormando, George Mason University (GMU). Each mat was collected by hand while wading near the shore, placed in Whirl‐pack sampling bags with river water and transported the same day to the Algal Ecology Lab at GMU's Potomac Environmental Research and Education Center (Woodbridge, Virginia) in a cooler on ice.

### 2.2 Sample Processing

Upon returning to the laboratory, both mat samples collected in 2024 and 2025 were processed using identical protocols to ensure that molecular and morphological data were derived from the same target taxon. Although each field sample contained a single *Limnofasciculus* mat, we accounted for the complexity of cyanobacterial mats, which typically comprise multiple species. Samples were first examined under a dissecting microscope at 50× magnification. Small portions of *Limnofasciculus* filaments were isolated from the same mat and transferred to a separate container, where they were carefully cleaned using fine forceps to remove visible *Microseira wollei* (Farloe ex Gomont) G.B. McGregor & Sendall ex Kenins and Microcoleus filaments present in both samples. Cleaner *Limnofasciculus* subsamples were then obtained by selecting small clusters of bright blue‐green filaments with consistent cell morphology, connected by mucilage in fascicles from the same mat. These subsamples were extracted from the same mat and were representative for the same organism. Subsamples designated for DNA extraction were placed in sterile 1.5 mL Eppendorf microtubes after excess water removal and stored at −80°C. Corresponding subsamples for morphological analysis were examined immediately in fresh condition. Additionally, a subsample of *Limnofasciculus* filaments from site NF11 (Sample 1) was preserved in 15 mL centrifuge tubes containing distilled water for toxin analysis and stored at −20°C. This approach ensured that molecular and morphological data were obtained from the same specimen and corresponded to the same target taxon, which was essential given that the study was based on field‐collected material.

### 2.3 Light Microscopy

Representative subsamples from the fresh field mats, which corresponded to the subsamples selected for molecular analysis, were observed and documented with an Olympus microscope BX41 with an SC30 digital camera (Olympus Imaging America, Center Valley, Pennsylvania, USA) and a Leica microscope BM4 B with a K3C digital camera (Leica Microsystems, Buffalo Grove, Illinois, USA) at magnification ×400 and ×1000 with oil immersion. We recorded morphological features such as cell width and length, colour, granulation, thylakoid position and extracellular mucilage. The size ranges given in the description of the cyanobacterial species are based on a minimum of 60 cells measured from 30 trichomes using the fresh specimen from the South Fork (Sample 2).

### 2.4 DNA Extraction

We used mat subsamples isolated from Sample 1 (2024) and Sample 2 (2025) as described above. Excess water was removed from 25 to 50 mg of filaments by pressing them between layers of KimWipes (Kimtech Science, Woodbridge, Ontario, Canada). The material was then transferred to ZR BashingBead Lysis Tubes (Zymo Research, Irvine, CA, USA) containing genome lysis buffer from a Quick DNA miniprep kit (Zymo Research). Bead beating was conducted for 10 min using a Vortex‐Genie 2 equipped with a Horizontal Microtube Holder attachment (Scientific Industries, Bohemia, NY, USA). After vortexing, the tubes were centrifuged briefly to remove foam and a small sample was removed for microscopic examination to ensure cell disruption had occurred. From this point forward, DNA extraction was carried out according to the manufacturer's instructions.

### 2.5 16S rRNA Amplicon and Shotgun Metagenomic Sequencing

From DNA extracted from both sampled mats (Sample 1 and Sample 2), the full‐length sequence of the small ribosomal RNA subunit (16S) was PCR‐amplified using the universal primers 27F (5′‐AGRGTTYGATYMTGGCTCAG‐3′) and 1492R (5′‐RGYTACCTTGTTACGACTT‐3′) published in Callahan et al. (2019), which are modified versions of those in Lane (1991). Amplification was carried out with Phusion DNA polymerase (ThermoFisher, Waltham, MA, USA) with the provided High Fidelity buffer, 5 pmol of each primer and a BioRad C1000 thermal cycler programmed to heat to 95°C, 10 min (95°C, 30 s, 55°C, 30 s, 72°C, 30 s) × 40 cycles, followed by a 72°C, 10 min soak. Amplification of a single amplicon was confirmed using gel electrophoresis, and the amplicon was purified for sequencing using the GeneJET PCR Purification kit (Thermo Fisher Scientific, Waltham, MA, USA). Sequencing of the amplicon was completed by the commercial sequencing facility, Genewiz (Azenta Life Sciences, North Plainfield, NJ, USA) using their PCR‐EZ Long‐Read Amplicon Sequencing service. DNA extracted from the South Fork mat subsample (Sample 2, 2025) was shotgun sequenced by Azenta using their non‐human NGS Illumina paired‐read sequencing service.

### 2.6 Sequenced Data Processing

To process the full‐length 16S sequence, the long‐read FASTQ file received from Azenta was imported into Geneious Prime v.2025.0 (Dotmatics, Boston, MA). Reads were de novo assembled using the Geneious assembler with a maximum mismatch threshold of 15%. Sixty‐seven percent of long reads assembled into a single sequence corresponding to 16S. A fulllength 16S sequence was also identified using the short‐read shotgun data. Paired‐read FASTQ files were imported into Geneious Prime and de novo assembled using the Geneious assembler. Genious' map to reference function was used to identify candidate 16S de novo contigs using the long‐read 16S sequence as bait. This approach identified a single 16S contig. Attempts to identify alternative contigs with 16S‐containing contigs using other reference sequences (e.g., *Microcoleus*) identified the same contig, confirming the 16S sequence using two strategies.

For whole genome analysis from the short‐read shotgun data, Illumina adapters and low‐quality reads were removed with *fastp v0.20.1* (Chen et al. 2018) using default settings. Cleaned reads were assembled into scaffolds with *metaSPAdes* (*SPAdes v3.12.0*) (Bankevich et al. 2012) using k‐mer options ‐k21,33,55,77. Metagenome‐assembled genomes (MAGs) were recovered from the assembly using the *variational autoencoders for metagenomic binning* pipeline (*VAMB v3.0.2*) (Nissen et al. 2021), *MetaBAT2 v2.15–6* (Kang et al. 2015) and *MaxBin v2.2.7* (Wu et al. 2016). We used *DasTool v1.1.4* (Sieber et al. 2018) to recover the highest‐quality non‐redundant MAGs from each assembly. MAG quality was estimated with *CheckM v1.1.3* (Parks et al. 2015). Taxonomic classification of MAGs was performed with *GTDB‐Tk v2.3.2* (Chaumeil et al. 2022) implemented in the *US Department of Energy Systems Biology Knowledgebase* (*KBase*) platform (http://www.kbase.us/) (Chivian et al. 2023). *GTDB‐Tk* makes use of the Genome Database Taxonomy (GTDB) r220 (Parks et al. 2022).

### 2.7 16S‐Based Phylogenetic Analysis

For 16S‐based phylogenetic analysis, the obtained full‐length sequence from our sampled mats was aligned to other cyanobacterial 16S sequences available in NCBI/Genbank (https://www.ncbi.nlm.nih.gov) as of July 2025. Sequences were aligned using MUSCLE using the PPP standard algorithm (Edgar 2004). A maximum likelihood tree was produced using *IQTREE v.3.0.1* (Trifinopoulos et al. 2016) using the HKY model and ultrafast bootstrapping (Hoang et al. 2018).

### 2.8 Whole Genome Phylogenetics and Genome‐Based Species Delimitation

Open reading frames (ORFs) were predicted from the assembled cyanobacterial MAG (cMAG) and reference genomes using *Prodigal v2.6.3* (Hyatt et al. 2010). Reference genomes classified within the family Coleofasciculaceae were retrieved from the GenBank database (accessed 7 December 2025) and complemented with Coleofasciculaceae MAGs previously described from aquatic systems in Atlantic Canada (Valadez‐Cano, Reyes‐Prieto, et al. 2025; Valadez‐Cano, Tromas, et al. 2025). For these previously published MAGs, raw sequencing data were retrieved and processed as described above. Only genomes with estimated completeness > 80% and contamination < 2% were retained for downstream analyses.

Whole‐genome phylogenetic reconstruction of Coleofasciculaceae genomes was performed using a single‐copy ortholog approach. Briefly, *OrthoFinder v2.5.5* (Emms and Kelly 2015, 2019) was used to identify single‐copy orthogroups, which were aligned and concatenated at the protein level using *MAFFT v7.490* (Katoh and Standley 2013). A maximum‐likelihood (ML) phylogeny was then inferred from the concatenated multiple sequence alignment using *IQ‐TREE v2.3.2* (Minh et al. 2020), applying the Q.plant + F + R8 substitution model selected by *ModelFinder* (Kalyaanamoorthy et al. 2017). Branch support was assessed using 2000 ultrafast bootstrap replicates. The tree was rooted with the genome of *Microcoleus* sp. GLPC (family Microcoleaceae) as the outgroup (Valadez‐Cano, Reyes‐Prieto, et al. 2025).

Average amino acid identity (AAI) between genomes was calculated using *FastAAI* (Gerhardt et al. 2025). Average nucleotide identity (ANI) and digital DNA–DNA hybridization (dDDH) values were estimated using *FastANI v1.33* (Jain et al. 2018) and the Genome‐to‐Genome Distance Calculator (GGDC) web service hosted by DSMZ (Meier‐Kolthoff et al. 2013), respectively. Thresholds of 95% ANI and 70% dDDH were applied for bacterial species delineation, following established criteria (Meier‐Kolthoff et al. 2013; Richter and Rosselló‐Móra 2009).

### 2.9 Comparative Genome Analysis

Protein sequences from the recovered cMAG and *Limnofasciculus baicalensis* strain BBK‐W‐15 were functionally annotated against the KEGG database using the online tool *BlastKOALA* using the ‘Prokaryotes’ reference genes dataset (Kanehisa et al. 2016; Kanehisa and Goto 2000).

BGCs were predicted using the bacterial version of the online antiSMASH platform (Blin et al. 2021). Predicted BGCs were subsequently parsed and summarized using *multiSMASH v0.4.0* (Zreitz 2024).

### 2.10 Toxin Analysis

We analysed Sample 1 (site NF11, North Fork Shenandoah River near Strasburg collected on 12 June 2024) used for DNA extraction and 16S‐based phylogenetic analysis and two additional samples, both collected on 26 June 2024: Sample SF2 (South Fork Shenandoah River at Grove Hill Boat Ramp; 38.52765, −78.59459) and Sample SF3 (South Fork Shenandoah River at Riverside Park at Elkton; 38.39648, −78.62447).

Algal material was extracted and analysed based on Aparicio‐Muriana et al. (2022). In brief, the algal material was freeze dried (Labconco 6‐L Freeze Dryer, Kansas City, Missouri, USA) and about 100 mg were mixed with 5% formic acid, homogenized (ULTRA‐TURRAX T25, IKA, Wilmington, North Carolina, USA) and centrifuged (9000 rpm, 10 min). The pellet was extracted a second time with water:methanol 20:80 v/v with sonication (20 min). After centrifugation, the extracts were combined, diluted to 25 mL with Milli‐Q water and extracted by solid‐phase extraction (Strata‐X and Oasis MCX SPE tandem cartridges). The toxin extracts were eluted with 5% NH_4_OH in methanol, filtered (0.20 μm PTFE), evaporated to dryness and reconstituted in 3% formic acid in acetonitrile. LC–MS/MS. Toxin analysis was performed by LC–MS/MS using a Shimadzu 8050 LCMS (Shimadzu, Columbia, Maryland, USA) using a SeQuant ZIC‐HILIC UHPLC column (150 mm × 2.1 mm, 3.5 μm, MilliporeSigma, St Louis, Missouri, USA) and binary mobile phase 0.3% formic acid and acetonitrile.

The samples were analysed for anatoxin‐a, homoanatoxin‐a, dihydroanatoxin‐a, saxitoxin, microcystin‐LR, microcystin‐RR, nodularin, cylindrospermopsin.

## 3 Results

### 3.1 16S rRNA Phylogeny and Genome‐Based Definition of the Novel Species *L. delicatus*

Analysis of full‐length 16S sequences from both specimens of *Limnofasciculus* from the North and South Forks of the Shenandoah River revealed that they differed from one another by a single nucleotide and thus represent a very closely related taxon. Phylogenetic analyses using the full length 16S gene demonstrated that both sequences were closely related to *L. baicalensis*, from which they differed by 9 (South Fork specimen) and 10 (North Fork specimen) nucleotides across 1421 bp (Figure S1).

Given the high conservation of 16S rRNA sequences between the Shenandoah River samples and *L. baicalensis* and the limited resolution of this marker for distinguishing closely related species, we employed a whole‐genome approach, which provides a more robust framework for species delimitation in cyanobacteria, including the use of high‐quality MAGs (Dvořák et al. 2025). From shotgun data from the South Fork sample, we recovered a near‐complete cyanobacterial metagenome‐assembled genome (cMAG) with an estimated completeness of 97.8% and 0% contamination. The assembled cMAG was 6,784,069 bp in length with a GC content of 42%, values that are highly similar to the previously reported genome size (6,604,967 bp) and GC content (42%) of *L. baicalensis* strain BBK‐W‐15 (Sorokovikova et al. 2023). GTDB‐Tk classified the recovered cMAG at the genus level as *Limnofasciculus* but could not be assigned to *L. baicalensis* at the species level.

**FIGURE 1:**
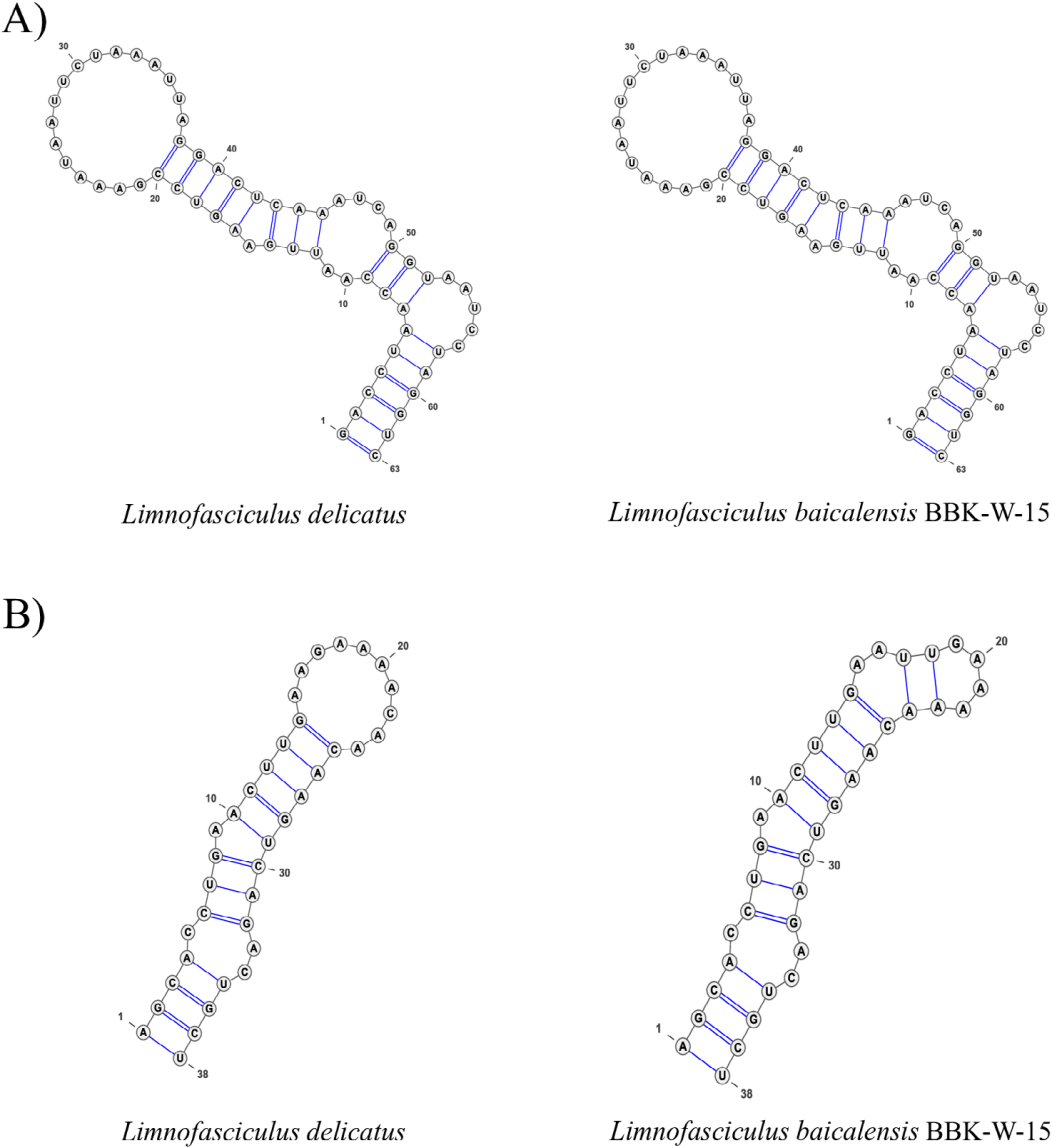
Comparison of (A) D1–D1′ and (B) Box B secondary structures from *Limnofasciculus delicatus* from the South Fork of Shenandoah River and *Limnofasciculus baicalensis* BBK‐W‐15 derived from the 16S–23S rRNA internal transcribed spacer (ITS) region. ITS motifs were identified using the Cyanobacterial ITS Motif Slicer (CIMS, Labrada et al. 2024) and RNA secondary structures were predicted using the IPknot web server (Sato et al. 2011).

From the recovered *Limnofasciculus* MAG we obtained a single 16S sequence showing identity > 99.9% across 1421 nucleotides with the sequences from the South Fork and North Fork, indicating that the sequences and the cMAG correspond to the same taxon across both sampling sites. Consistent with the high identity of the 16S between the *Limnofasciculus* from the Shenandoah River and *L. baicalensis* BBK‐W‐15, the D1–D1′ helices predicted from the 16S–23S ITS regions were identical in both taxa, resulting in identical secondary structures (Figure 1A). In contrast, comparison of the Box B helices revealed sequence divergence that produced clear structural differences between the two taxa (Figure 1B). The Box B regions shared 89.7% pairwise identity and similar basal regions but differed in their terminal loops: four substitutions in *L. baicalensis* reduced the terminal loop to four nucleotides, compared with the 10‐nucleotide loop observed in the *Limnofasciculus* specimen from South Fork (Figure 1B).

**FIGURE 2:**
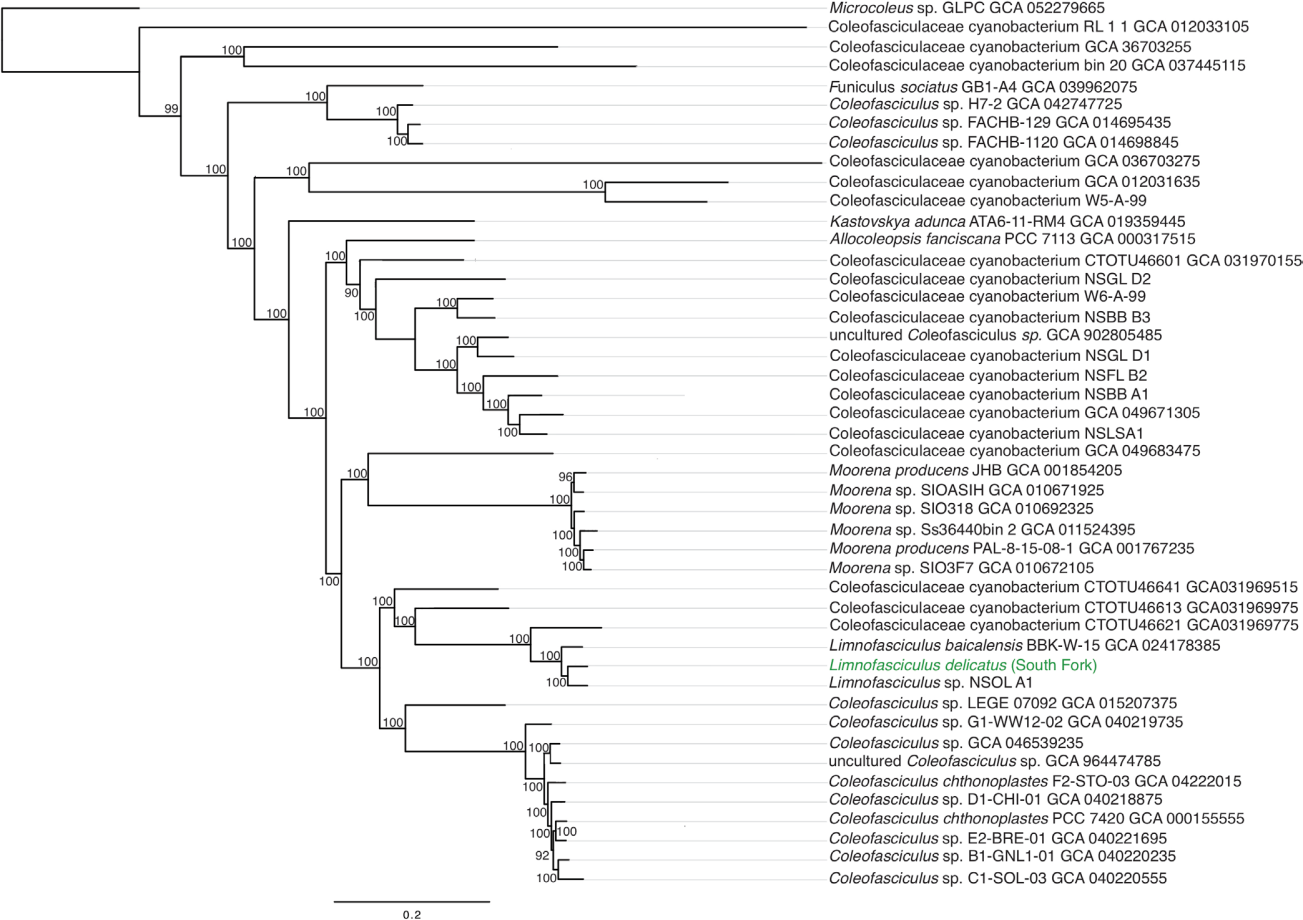
Maximum likelihood phylogeny estimated using the Q.plant + F + R8 substitution model based on a concatenated alignment (data set available upon request) of 1085 orthogroups comprising 313,222 amino acid sites from *Limnofasciculus delicatus* from the South Fork of Shenandoah River and other Coleofasciculaceae genomes. Bootstrap support values ≥ 90% are indicated at the nodes. The scale bar represents amino acid substitutions per site. The tree was rooted arbitrarily using *Microcoleus* sp. GLPC (Microcoleaceae) as outgroup.

Consistent with the 16S rRNA phylogeny, whole‐genome ML analysis placed the *Limnofasciculus* MAG in a well‐supported clade (100% bootstrap support) together with *L. baicalensis* BBK‐W‐15 and *Limnofasciculus* sp. NSOLA1, recently described from Oathill Lake in Nova Scotia, Canada (Valadez‐Cano, Tromas, et al. 2025) (Figure 2). This *Limnofasciculus* clade also included multiple unclassified Coleofasciculaceae genomes and was clearly distinct from a separate clade containing *Coleofasciculus chthonoplastes* (Gomont) M. Siegesmund, which was likewise strongly supported (100% bootstrap; Figure 2).

AAI estimates between the recovered *Limnofasciculus* MAG, *L. baicalensis* BBK‐W‐15 and *Limnofasciculus* sp. NSOLA1 were > 81%, whereas AAI values with all other reference genomes were < 73%. This pattern is consistent with the proposed 60%–80% AAI threshold for genus delineation (Konstantinidis and Tiedje 2005; Rodriguez‐R and Konstantinidis 2014).

To assess species‐level boundaries, we further calculated ANI and dDDH metrics. The *Limnofasciculus* MAG from the Shenandoah River showed ANI values of 91% and 90% with *Limnofasciculus* sp. NSOLA1 and *L. baicalensis* BBK‐W‐15, respectively, and < 84% with all other reference genomes. Also, the dDDH metrics were below 50% between each of the *Limnofasciculus* genomes analysed. These genome‐wide relatedness values fall far below the commonly accepted thresholds of 95% ANI and 70% dDDH for bacterial species delimitation.

Collectively, these genome‐based analyses support the assignment of *Limnofasciculus* sp. NSOLA1, *L. baicalensis* and the *Limnofasciculus* MAG from the Shenandoah River to the genus *Limnofasciculus* while recognizing them as three distinct species.

Based on the combined phylogenetic and genomic evidence which is consistent with the recent cyanobacterial taxonomy frameworks (e.g., Dvořák et al. 2025), we formally describe this benthic cyanobacterium collected from the South Fork of the Shenandoah River in 2025 as a new species *Limnofasciculus delicatus* Stancheva, Cahoon & Valadez‐Cano.

### 3.2 Comparative Genomic Analysis of *Limnofasciculus* Species

**FIGURE 3:**
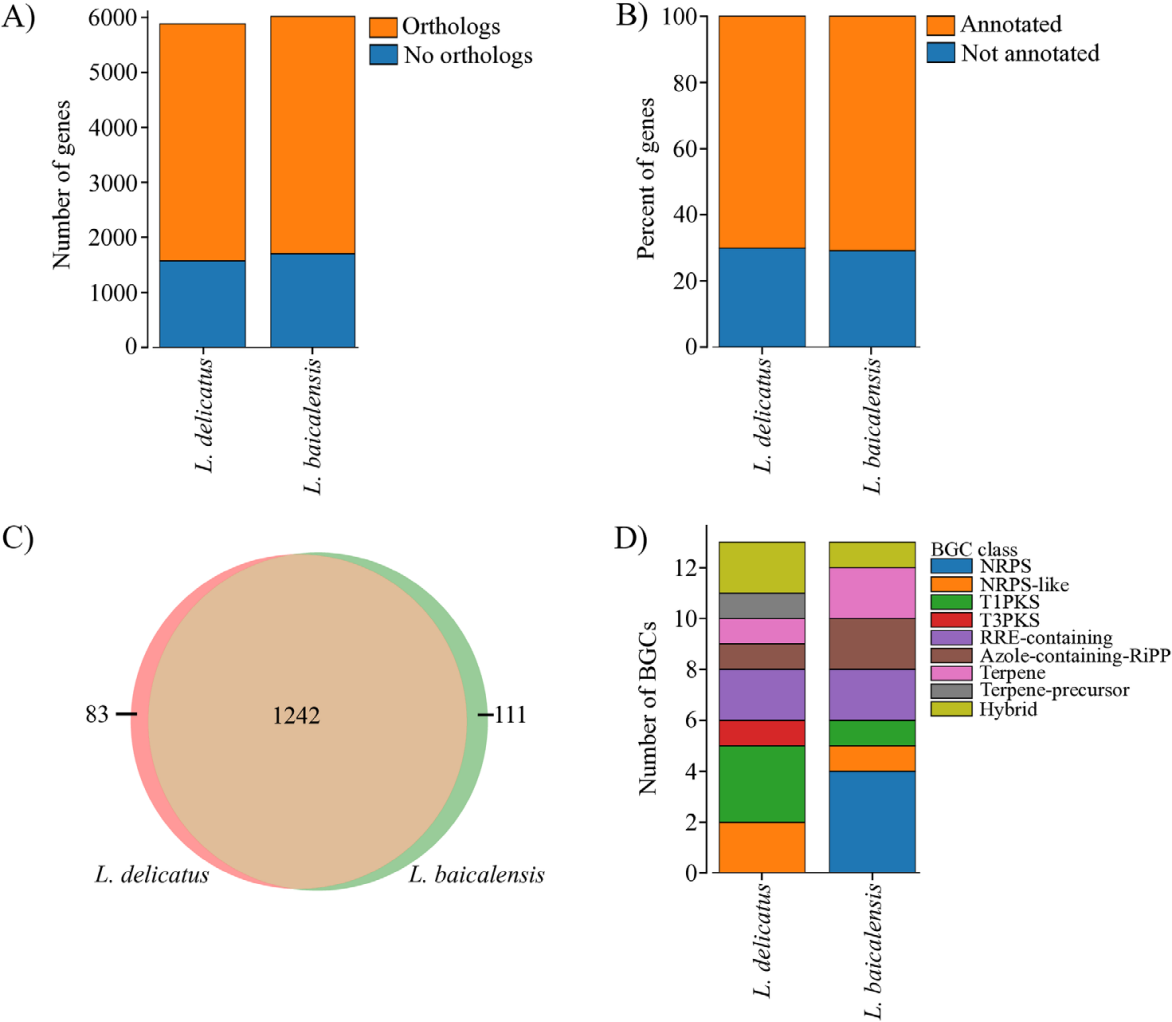
Comparative genomic analysis of *Limnofasciculus delicatus* from the South Fork of Shenandoah River and *Limnofasciculus baicalensis* BBK‐W‐15. (A) Ortholog‐based comparison of protein‐coding genes between both genomes, showing shared and species‐specific gene content. (B) Percent of protein‐coding genes assigned to KEGG orthology (KO) functions in each genome. (C) Distribution of KEGG orthologs, highlighting shared core functions and functions unique to each species. (D) Biosynthetic gene cluster (BGC) composition identified in each genome.

A total of 5880 protein‐coding sequences were predicted in *L. delicatus* , of which 4307 (73.2%) had an identifiable ortholog in *L. baicalensis* BBK‐W‐15 (Figure 3A). Conversely, *L. baicalensis* BBK‐W‐15 encoded 6015 protein‐coding sequences, with 4313 (71.7%) sharing orthologs with *L. delicatus* (Figure 3A). Approximately 30% of the coded proteins in each genome were assigned to KEGG orthology (KO) functions (Figure 3B). Among these annotated functions, 1242 KEGG orthologs were shared between the two species, representing core functional annotations, whereas 83 KEGG functions were unique to *L. delicatus* and 111 were unique to *L. baicalensis* BBK‐W‐15 (Figure 3C).

To characterize the environmental adaptations of *L. delicatus* we examined for *nif* genes related to nitrogen fixation and *gvp* genes for gas vesicle formation, which are missing and the biosynthetic pathway for thiamine biosynthesis which was incomplete. Furthermore, we did not identify any BGCs associated with the production of known cyanotoxins in either genome. Both species harboured 13 predicted BGCs; however, their composition differed markedly (Figure 3D). *L. delicatus* was enriched in Type I polyketide synthase (T1PKS) and hybrid clusters, whereas *L. baicalensis* BBK‐W‐15 exhibited a higher prevalence of nonribosomal peptide synthetase (NRPS) and terpene‐associated clusters (Figure 3D). The proportion of the genome dedicated to secondary metabolite biosynthesis was also higher in *L. delicatus* (6%) than in *L. baicalensis* BBK‐W‐15 (3.2%). Collectively, these differences point to distinct secondary metabolite biosynthetic potentials despite similar overall BGC counts.

### 3.3 Toxin Analysis

All three samples tested were negative for all toxins, except for sample SF2, which tested positive for homoanatoxin‐a (3.65 ng/mg). However, microscopic analysis showed the presence of *Microcoleus* and *Phormidium* filaments within *Limnofasciculus* mats (Figure S2), which are likely to be responsible for homoanatoxin‐a detection.

### 3.4 Species Description

#### 3.4.1 *Limnofasciculus delicatus* Stancheva, Cahoon & Valadez‐Cano, sp. nov. (Figures 4, 5, 6, 7)

**FIGURE 4:**
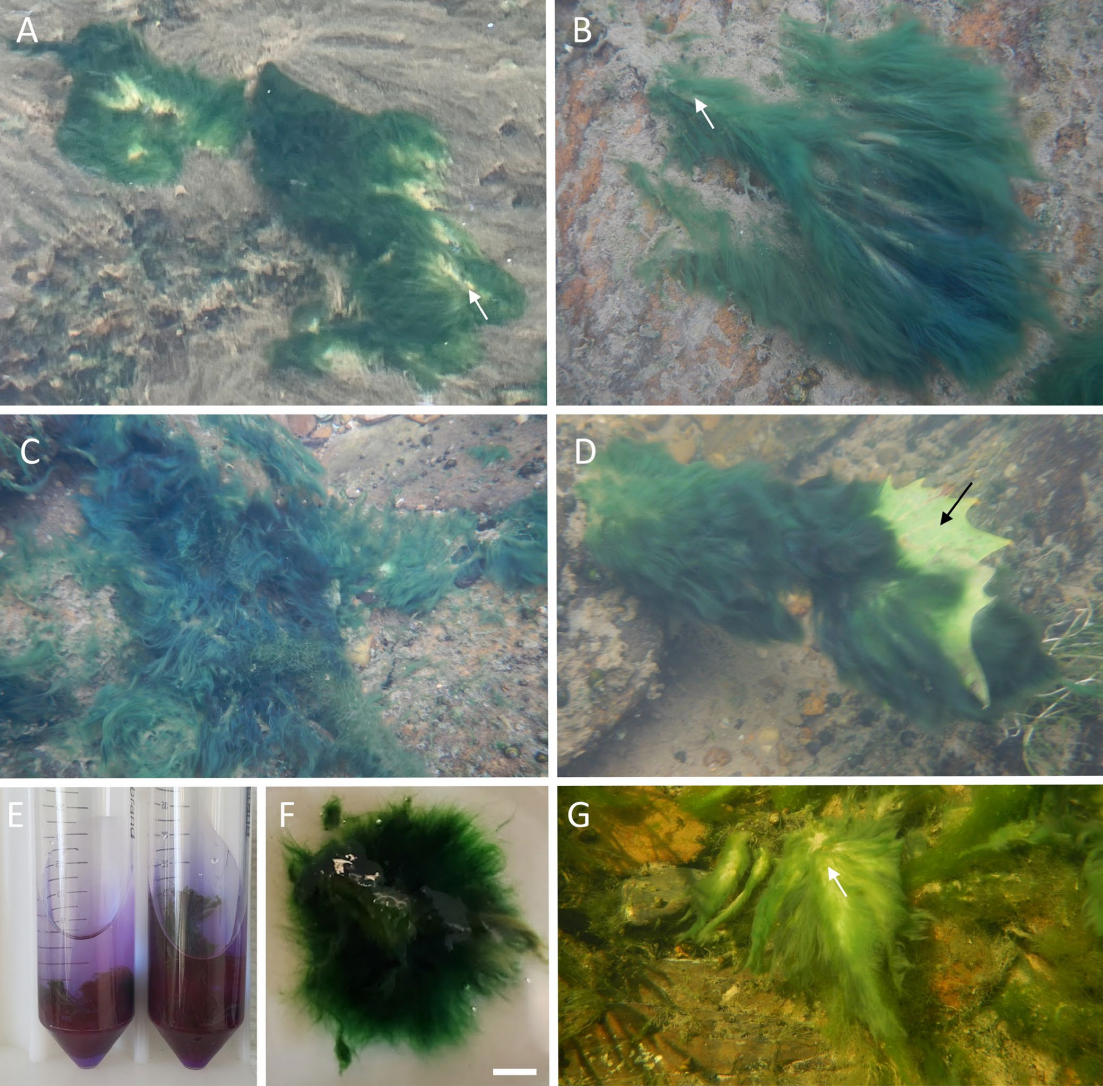
Field view of *Limnofasciculus delicatus* mats from Shenandoah River. Mats are blue‐green (A–D, G), with branched bushy appearance, attached by extensive colourless mucilage at one end (white arrows). Some mats appear more bluish due to leaking of phycocyanin from the cells in the field (C) and in lab conditions (E). Black arrow (D) indicates tree leaf trapped within mats. Single mat in a petri dish in lab conditions (F). Images A and E are from the sequenced specimen from the type locality South Fork Shenandoah River upstream of Front Royal. Scale bar for (F) = 2 cm.

**FIGURE 5:**
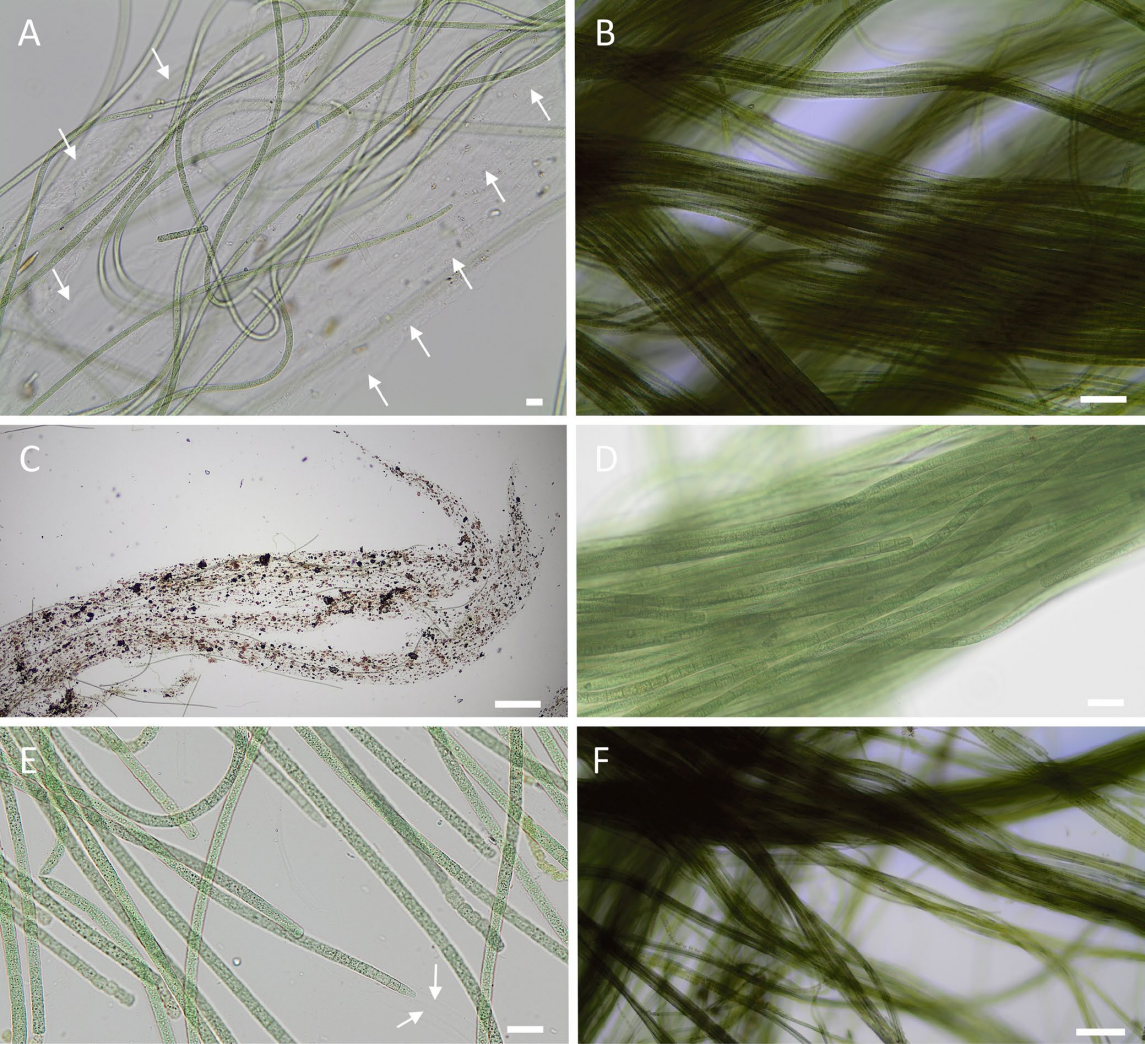
Low magnification light micrographs of *Limnofasciculus delicatus* mats from Shenandoah River. Mats are composed of many fascicles enclosed in a common sheath (B, F). A fascicle is a bundle with many parallel filaments (D) joined together by agglutinating colourless individual sheaths (arrows on E) and common sheaths (arrows on A) hard to distinguish. The colourless common sheath is best visible when colonized by diatoms in the bottom parts of the mats (C); the sheath is bifurcate and tapering at the tips (C). Images (C) and (E) are from the sequenced specimen from the type locality South Fork Shenandoah River upstream of Front Royal. Scale bar = 20 μm (A, D, E) and 100 μm (B, C, F).

**FIGURE 6:**
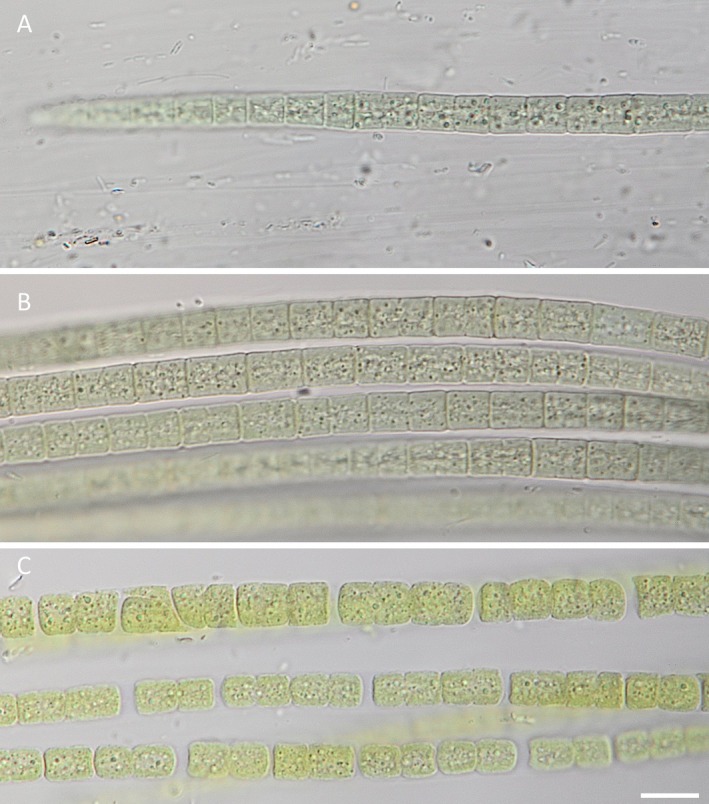
High magnification light micrographs of *Limnofasciulus delicatus* filaments from Shenandoah River. A filament enclosed in widened and lamellated colourless common sheath (A). Fascicle with parallel filaments joined together by agglutinating colourless common sheaths hard to distinguish (B). Filaments with ruptured and detached cells due to lab conditions (added distilled water and room temperature) release water‐soluble pigment phycocyanin (C). All images are from the sequenced specimen from the type locality South Fork Shenandoah River upstream of Front Royal. Images obtained by differential interference contrast light microscopy, at ×1000 magnification and immersion oil. Scale bar = 10 μm (A–C).

**FIGURE 7:**
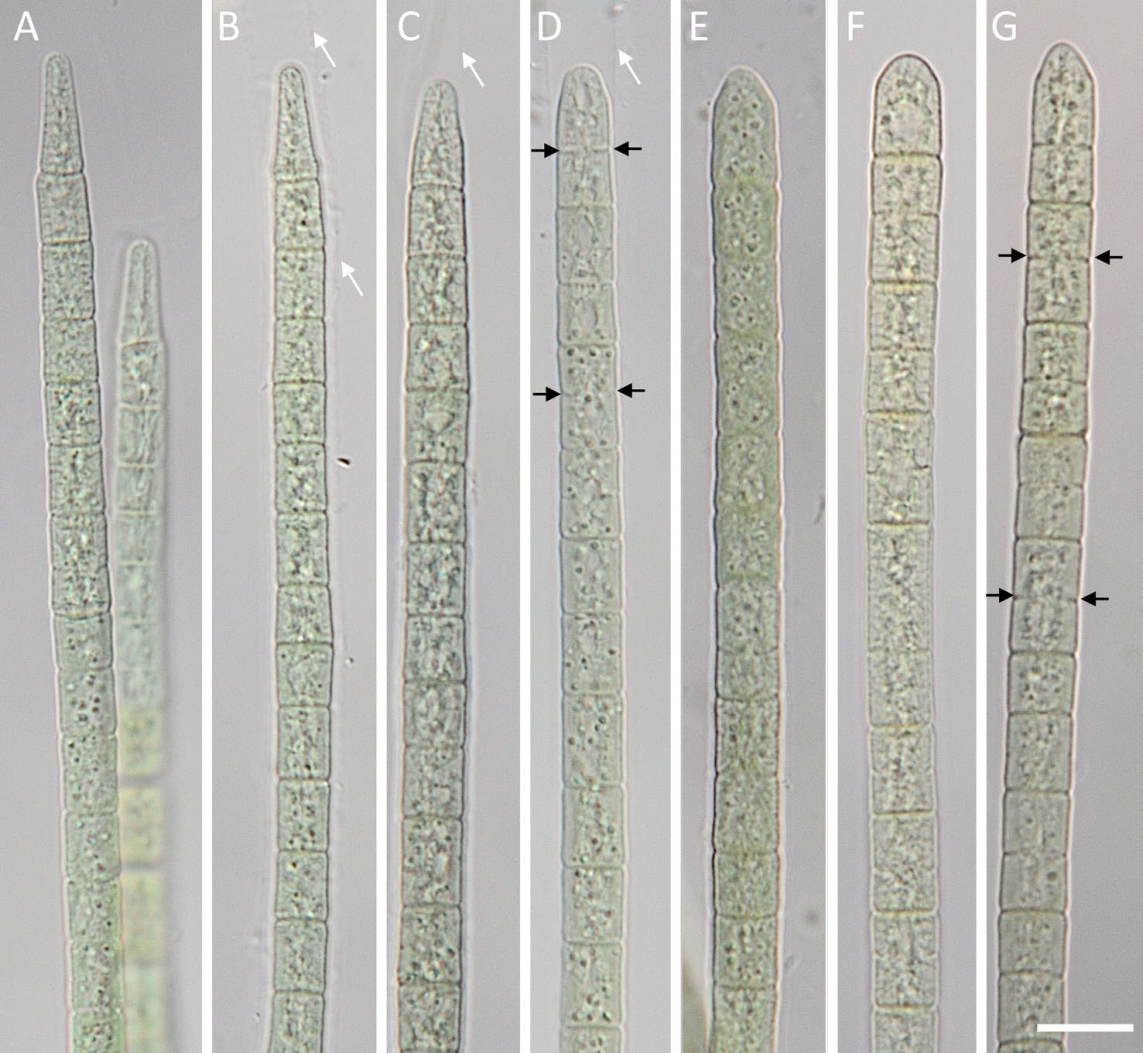
High magnification light micrographs of *Limnofasciculus delicatus* filaments from Shenandoah River showing variation in cell morphology (A–G). Cells are quadrate to long cylindrical with distinct constrictions at the cross‐walls (A–G) and intracellular granulation (A, B, D); cell division commonly observed (black arrows on D and G). Apical cells without calyptra, bullet‐shaped, longer than wide, with variable degree of tapering of the tip from sharp (A—C) to more conical rounded (D—G); Individual sheath around each trichome (white arrows) is usually thin (D), rarely thick, diffluent at the margin (C) or with fine transverse folds (B). All images are from the sequenced specimen from the type locality South Fork Shenandoah River upstream of Front Royal. Holotype specimen (D). Images obtained by differential interference contrast light microscopy, at ×1000 magnification and immersion oil. Scale bar = 10 μm (A–C).

*Macroscopic view of the mats in the field*: Thallus is dark emerald/blue‐green, up to 40 cm long, approximately 5–25 cm wide, soft to touch, thick, fluffy, with long bushy (tuft‐like) pointed fasciculate outgrowths freely oscillating in the water current (Figure 4A–D,F,G). Large patches have a branched appearance due to their fasciculate structure and are attached by extensive colourless mucilage at one end. The mucilaginous attachment layer may look yellowish due to the accumulation of diatoms and sediment particles (Figure 4B,G). Large mats are typically attached to rocks and gravel and may cover large areas of the hard stream bottom. However, when the hard bottom substrate is colonized by aquatic macrophytes, *Limnofasciculus* mats are observed trapped within the vegetation.

*Microscopic view*: Thallus has a branched or bushy appearance because it is composed of many fascicles (bundles of filaments) enclosed in a common sheath (Figure 5B,F). Fascicles contain many parallel filaments, sometimes up to 30 or more (Figures 5D and 7B), intertwined or joined together by agglutinating individual (Figure 5E) and common sheaths (Figures 5A and 7A), often bifurcate at the tips (Figure 5C). Sheaths are colourless, open at the apical ends. Individual sheaths around each trichome are thin, smooth and firm (Figure 7D), rarely thick, diffluent at the margin (Figure 7C) or with fine transverse folds (Figure 7B). The individual sheaths agglutinate in a common widened and lamellated sheath (Figure 6A) that covers the fascicles and tapers to the top of the mats (Figure 5C). The colourless sheath does not stain with Lugol's.

Trichomes are dark blue‐green, cylindrical, long, straight or slightly wavy, with basal‐distal differentiation tapering towards conical apex, with distinctly constricted cells at the cross‐walls and enclosed in an individual thin colourless sheath. Trichomes are unbranched, slightly gliding; hormogonia not observed. Cells are short to long cylindrical with distinct constrictions at the cross‐walls, 4.8–8.7 μm wide; 4.5–26.7 μm long; 0.67–3.06 length to width ratio (Figure 7). Cells with radial or fasciculate thylakoid arrangement and granular content (Figure 7A–D). Cell division is commonly observed (Figure 7D,G). Minimum and maximum lengths refer to cells that had recently divided or were about to divide. Apical cells are without calyptra or thickening, bullet‐shaped with a variable degree of tapering of the tip, always longer than wide (Figure 7), 4.15–7.4 μm wide; 7.9–15 μm long; 1.12–3.03 length to width ratio.

Cell wall is thin and easily ruptured under stress, such as changing osmotic pressure by adding distilled water or increased temperature in lab conditions. In this condition, cells lose their integrity, detach and release cellular content and water‐soluble pigment phycocyanin (Figures 4E and 6C). Similar bluish colour of the fascicles was observed in the field material (Figure 4C), indicating senescing cells or filament degradation under unfavourable environmental conditions.

*Holotype voucher specimen*: Glutaraldehyde preserved specimen UC2110787 deposited in the University Herbarium at University of California, Berkeley. Holotype specimen is illustrated on Figure 7D.

*Type locality*: South Fork Shenandoah River upstream of Front Royal (38.86766, −78.28067). The sequenced specimen was collected on 13 August 2025, by Jacob Mormando.

*GenBank Bioproject #*: PRJNA1419196.

*Etymology*: The species epithet, meaning *delicate*, *tender* or *fine in texture*, reflects the thin cell walls and their unusually rapid degradation under stress conditions, leading to the quick extracellular release of blue, water‐soluble phycobilins.

*Taxonomic notes*: This aquatic cyanobacterium is morphologically most similar to two species with trichomes that have elongated cells with constricted cross walls and apical cells without thickening or calyptra that were previously classified as *Microcoleus*, for example, *M. chthonoplastes* Thuret ex Gomont (currently *Coleofasciculus chthonoplastes*) and *M. lacustris* Farlow ex Gomont, which also need reinvestigation.

*M. lacustris* differs by narrower trichomes and shorter apical cells (cells 3–5.7 μm wide; 4–14.5 μm long according to Komárek and Anagnostidis 2005), but descriptions in the literature are incomplete and photomicrographs are missing for reliable comparison. Since *M. lacustris* was described from a close geographical location (Massachusetts, Middlesex, Newton, USA) we made attempts to obtain the herbarium material (Farlow et al. 1889, catalogue numbers SD00007515 and Y.U095815) but it was not available for sequencing. *Limnofasciculus baicalensis* is geographically distant (benthic species in Lake Baikal, Russia) and morphologically different. The mats and cells are reddish; the trichomes are wider (7–11.6 μm according to Sorokovikova et al. 2023) and group in smaller fascicles with less than 10 trichomes in a common sheath.

*Environmental data*: Site NF11 (12 June 2024)—conductivity 404.2–436 μS/cm, salinity 0.19–0.21 PSU, pH 7.95–8.24, water temperature 18.09°C–19.97°C, dissolved oxygen 8.29–10.77 mg/L. Sites SF2 and SF3 (26 June 2024)– conductivity 386.8–408.1 μS/cm, salinity 0.18–0.19 PSU, pH 8.16–8.39, water temperature 23.26°C–25.03°C, dissolved oxygen 7.49–9.53 mg/L.

## 4 Discussion

Our study adds a second freshwater species to a recently described genus *Limnofasciculus* (Sorokovikova et al. 2023) and contributes to resolving taxonomic ambiguity within Coleofasciculaceae, a lineage undergoing rapid revision (Strunecký et al. 2023). Morphologically, both *Limnofasciculus* species have similar thallus and trichome characteristics, except for the strikingly different cell colour (red vs. cyan). The pigment composition in *Limnofasciculus* was not analysed by Sorokovikova et al. (2023) or in the present study; however, the observed differences in coloration are likely due to varying proportions of phycobiliproteins (phycocyanin and phycoerythrocyanin), as previously reported for several genera within the family *Coleofasciculaceae* (Fernandes et al. 2021).

Currently, there are 19 genera and 44 species within *Coleofasciculaceae* (Guiry and Guiry 2026) showing variable morphology of the trichomes and the extracellular polysaccharide sheaths. Yet, their trichomes are always unbranched, lack specialized cells such as heterocytes or spores and are composed of typically constricted cells with apical cells lacking a calyptra (Fernandes et al. 2021; Strunecký et al. 2023; Sorokovikova et al. 2023). Bushy colonies composed of fascicles with parallel trichomes in colourless sheath; blue‐green elongated cells with variable degree of constrictions at cell walls and conical to bullet‐shaped, tapering apical are morphological features shared between *Limnofasciculus delicatus* and other genera within Coleofasciculaceae, such as *Coleofasciculus* (Siegesmund et al. 2008), *Funiculus* (Fernandes et al. 2021), *Karukerafilum* (Halary et al. 2024), *Paludothrix* (Wu et al. 2024) and *Symbiothallus* (Chen et al. 2025). Despite some morphological similarities, these taxa have different ecology; they were described from marine habitats, mangroves ecosystems, soil crusts, wetlands and fungus‐cyanobacterium symbiosis from subtropical forest. Ecological and habitat characteristics play a critical role in cyanobacterial taxonomy (Johansen and Casamatta 2005). These ecological traits provide important complementary evidence to phylogenetic data and contribute significantly to species delimitation and taxonomic resolution in cyanobacteria (Komárek et al. 2014; Komárek 2016).

The genus *Limnofasciculatus* is reported from freshwater aquatic ecosystems only—the Lake Baikal (Sorokovikova et al. 2023) and flowing waters in Northern Virginia, USA (this study). The recovery of *L. delicatus* in the North Fork of the Shenandoah River in 2024 and in the South Fork of the Shenandoah River in 2025, despite their spatial separation and temporal offset, indicates that the benthic cyanobacterium is not a transient or episodic occurrence but rather a persistent component of the riverine microbial community. This temporal and geographical continuity suggests that *L. delicatus* is well adapted to the environmental conditions of the Shenandoah River and capable of maintaining stable populations across seasons and river segments.

Our findings expand the known ecological breadth of this recently described genus. In contrast to *L. baicalensis* (Sorokovikova et al. 2023) and *Limnofasciculus* sp. NSOLA‐1 (Valadez‐Cano, Reyes‐Prieto, et al. 2025; Valadez‐Cano, Tromas, et al. 2025), which have been identified in lentic environments, *L. delicatus* was consistently recovered from a lotic system. This distinction suggests that members of the genus occupy a broader range of hydrological niches than previously recognized and that adaptation to flowing‐water environments has likely occurred within *Limnofasciculus*. Our analyses further highlight ecological differentiation among closely related filamentous cyanobacteria, as *Limnofasciculus* species have thus far been observed in freshwater environments, whereas the related genus *Coleofasciculus* has been predominantly reported from marine microbial mats (Siegesmund et al. 2008; Marter et al. 2025). Although additional sampling is required to robustly resolve ecological boundaries between these genera, current evidence points to divergent habitat specialization.

Despite this ecological differentiation, *L. delicatus* and *L. baicalensis* exhibited high 16S rRNA gene sequence similarity (99.3%), a value that falls within the range commonly observed among members of the same bacterial species (Hackmann 2025). However, species delimitation based solely on 16S rRNA gene similarity relies on lineage‐specific thresholds that should be regarded as guideline values rather than fixed criteria (Dvořák et al. 2023). Accordingly, whole‐genome–based species delimitation using ANI and dDDH clearly supports the classification of these taxa as distinct species when evaluated against established genomic thresholds. According to the current concept of cyanobacterial taxonomy (Dvořák et al. 2025; Riesco and Trujillo 2024), 95% ANI and 70% dDDH are a standard species delineation threshold. While the 16S rRNA gene remains a valuable marker for higher‐level phylogenetic placement, its conservative evolutionary rate limits its resolving power at the species level (Jain et al. 2018). In contrast, genome‐wide metrics such as ANI and dDDH capture variations across the entire genome and provide substantially higher resolution among closely related taxa (Jain et al. 2018).

The results of the secondary structures of the D1–D1′ helix and Box‐B helix from the 16 S–23 S ITS region showed that the D1–D1′ helices were identical in both *Limnofasciculus* taxa, while the Box B helices revealed sequence divergence that produced clear structural differences in their terminal loops: four substitutions in *L. baicalensis* reduced the terminal loop to four nucleotides, compared with the 10‐nucleotide loop observed the *L. delicatus* . Similarly, the analysis of the secondary structures of the 16 S–23 S ITS region in recently described *Microcoleus anatoxicus* Stancheva & K. Y. Conklin from California (USA) showed a single nucleotide difference in D1–D1′ helix and no nucleotide differences in the Box‐B helix compared to the genetically closest *Microcoleus* strain from New Zealand (Conklin et al. 2020). Due to difficulties using ITS rRNA regions for taxonomy purposes discussed by Villanueva et al. (2024) we used the secondary structure dissimilarity comparison only as an optional supporting criterion for species delineation, as recommended by Dvořák et al. (2025). However, our study contributed data that can be applicable in future use of ITS variation in relation to interspecific divergence within *Limnofasciculus*. For example, Siegesmund et al. (2008), who analysed a large dataset of laboratory cultures of *Coleofasciculus*, concluded that ITS secondary structure was congruent with 16S rRNA gene phylogenies and helpful in revealing additional evolutionary diversity within the species cluster of *C. chthonoplastes*.

Comparative genomic analyses further revealed that approximately 70% of the genomes of *L. delicatus* and *L. baicalensis* are shared and composed largely of highly conserved functional genes, indicating the presence of a substantial core genome within the genus. Despite this high degree of genomic conservation, the two species differed markedly in their predicted secondary metabolite biosynthetic potential. Notably, *L. delicatus* was predicted to allocate approximately twice the genomic fraction to secondary metabolite biosynthesis compared to *L. baicalensis* and the composition of predicted secondary metabolite gene clusters differed substantially between the two species. Given the well‐established ecological roles of secondary metabolites in mediating microbial interactions, competition and habitat adaptation (Vining 2007), these differences suggest that the two species produce distinct suites of metabolites with potentially divergent ecological functions.

No BGCs associated with the production of known cyanotoxins were identified in any *Limnofasciculus* genome analysed, which is consistent with the absence of anatoxin detection in most samples, with exception of sample SF2. Anatoxins detection in mats dominated by non‐toxigenic cyanobacteria has been reported previously and attributed to the persistence of toxigenic species in the environment (Valadez‐Cano, Tromas, et al. 2025). Similarly, trace levels of anatoxin‐a were detected in field mats dominated by the saxitoxin‐producing cyanobacterium *Microseira wollei*, but their source was uncertain and likely attributable to other cyanobacteria present within the mats (Smith et al. 2019). Although the toxigenic potential of *Limnofasciculus* cannot be ruled out and further sampling is required, its cooccurrence with anatoxin‐producing *Microcoleus* (Valadez‐Cano, Tromas, et al. 2025) may lead to misleading interpretations of anatoxin presence in *L. delicatus* ‐dominated mats if the full community composition is not considered.

Previous studies have suggested that anatoxin‐producing *Microcoleus* lineages exhibit genome streamlining and may depend on metabolic interactions with coexisting non‐toxigenic *Microcoleus* or phylogenetically distinct cyanobacteria (Tee et al. 2021; Valadez‐Cano, Reyes‐Prieto, et al. 2025; Valadez‐Cano, Tromas, et al. 2025). For example, mats producing homoanatoxin‐a were strongly associated with the presence of *Limnofasciculus* sp. NSOLA‐1 (Valadez‐Cano, Reyes‐Prieto, et al. 2025; Valadez‐Cano, Tromas, et al. 2025). Collectively, these observations suggest that members of the genus *Limnofasciculus* may play an indirect yet relevant role in anatoxin‐producing benthic mats, potentially through metabolic complementation or ecological facilitation, a hypothesis that warrants targeted experimental and genomic investigation. On the other hand, the interactions with *Microcoleus* and other mat‐invaders may not be beneficial for *Limnofascisulus* growth, as indicated by the presence of terpene‐associated clusters and azole‐containing RiPPs in its genome, which have distinct chemical defence roles helping compete for resources and protecting ecological niches from competitors (Martins and Vasconcelos 2015; Machado et al. 2025).

## Author Contributions

**Janice Lawrence:** conceptualization, investigation, writing – original draft, methodology, supervision. **Gordon M. Selckmann:** funding acquisition, writing – review and editing. **A. Bruce Cahoon:** conceptualization, investigation, funding acquisition, writing – original draft, methodology, writing – review and editing, formal analysis, supervision. **Rosalina Stancheva:** conceptualization, investigation, funding acquisition, writing – original draft, methodology, visualization, writing – review and editing, formal analysis, supervision. **Cecilio Valadez‐Cano:** conceptualization, investigation, writing – original draft, methodology, validation, visualization, writing – review and editing, formal analysis, data curation. **Benoit Van Aken:** formal analysis, writing – review and editing, funding acquisition.

## Funding

This research was funded in part by 4‐VA, a Collaborative Partnership for Advancing the Commonwealth of Virginia grant number (M13706), and the Virginia Interstate Commission on The Potomac River Basin (grant number: 224436) and VA Department of Environmental Quality as part of the project Investigation of Drivers of Harmful Algal Blooms on the Shenandoah River. Partial support was provided by research Grant # 2222322, which was awarded to Dr. Rosalina Stancheva (Co‐PI) by the United States National Science Foundation. We thank both anonymous reviewers and the editor Dr. Astha Nautiyal for the valuable comments which have helped us improve the presentation of our study.

## Conflicts of Interest

The authors declare no conflicts of interest.

## Supporting information


**Figure S1:** Maximum‐likelihood phylogeny based on 16S rRNA gene sequences of *Limnofasciculus delicatus* from the North (2014) and South Fork of Shenandoah River (2025) and representative cyanobacterial taxa.
**Figure S2:** Light micrographs of *Limnofasciculus delicatus* mat from Shenandoah River mixed with *Microcoleus* filament (arrows). Scale bar = 10 μm

## Data Availability

The data that support the findings of this study are available in GenBank at https://www.ncbi.nlm.nih.gov, reference number PZ400688. These data were derived from the following resources available in the public domain: Bioproject PRJNA1419196, https://www.ncbi.nlm.nih.gov/genbank/—PZ400688, https://www.ncbi.nlm.nih.gov.
