# Supplementary material for: Limnofasciculus delicatus (Coleofasciculaceae, Coleofasciculales), a Novel Mat‐Forming Cyanobacterium From Shenandoah River, Virginia, USA
Source: Environ Microbiol Rep. 2026 Jun 21;18(3):e70377. doi: 10.1111/1758-2229.70377 (PMC13283753; doi:10.1111/1758-2229.70377)
Supplement: Supplementary file 1 — Figure S1: Maximum‐likelihood phylogeny based on 16S rRNA gene sequences of Limnofasciculus delicatus from the North (2014) and South Fork of Shenandoah River (2025) and representative cyanobacterial taxa. Figure S2: Light micrographs of Limnofasciculus delicatus mat from Shenandoah River mixed with Microcoleus filament (arrows). Scale bar = 10 μm [file EMI4-18-e70377-s001.docx]

***Limnofasciculus delicatus* (Coleofasciculaceae, Coleofasciculales), a novel mat-forming cyanobacterium from Shenandoah River, Virginia, USA**

***Limnofasciculus delicatus sp. nov.* (Cyanobacteria)**

Rosalina Stancheva^1*#^, Cecilio Valadez-Cano^2#^, Benoit Van Aken^3^, Gordon M. Selckmann^4^, Janice Lawrence^2^, A. Bruce Cahoon^5^

Supplementary Figures


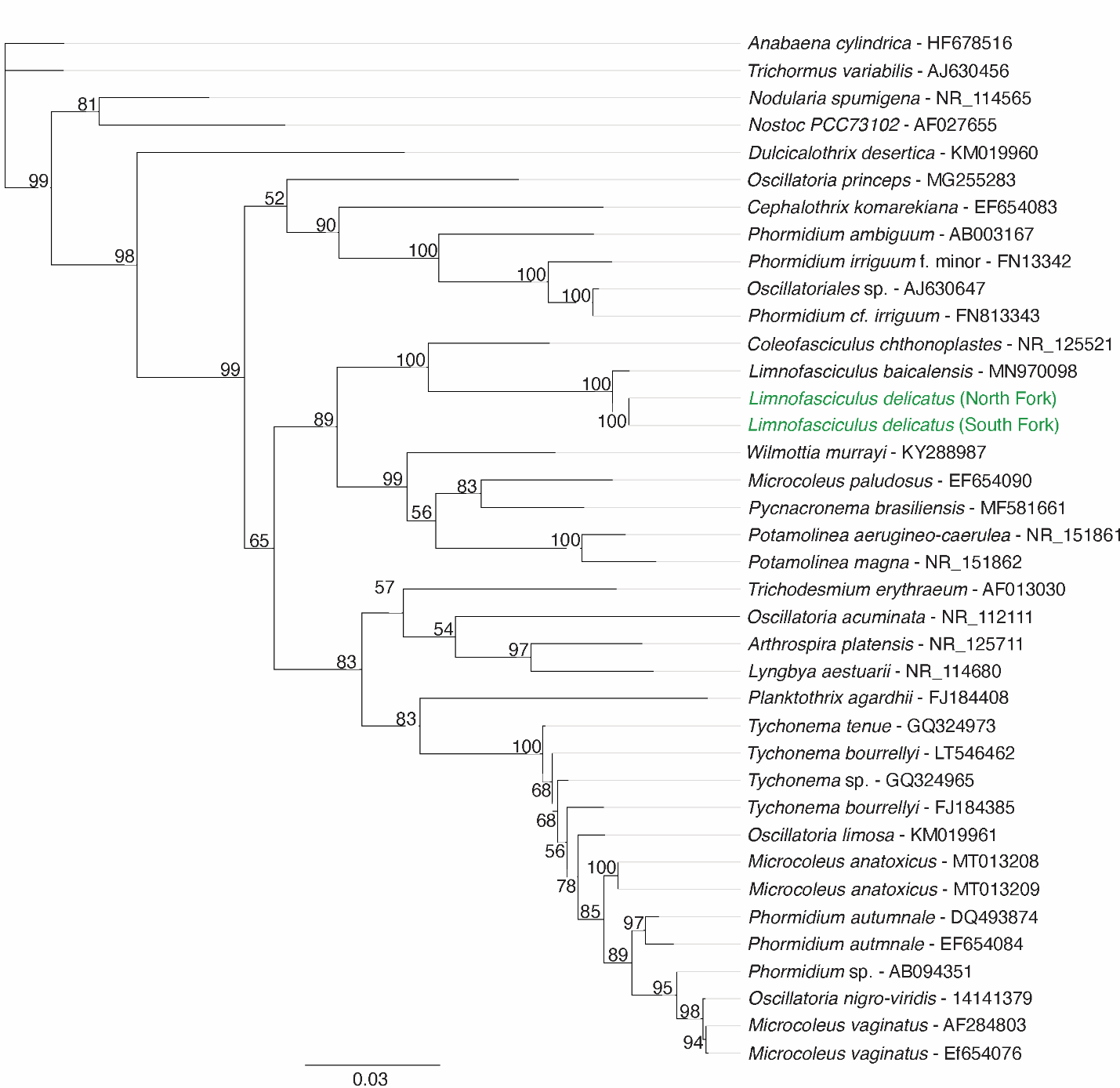


Supplementary Figure S1. Maximum-likelihood phylogeny based on 16S rRNA gene sequences of *Limnofasciculus delicatus* from the North (2014) and South Fork of Shenandoah River (2025) and representative cyanobacterial taxa.


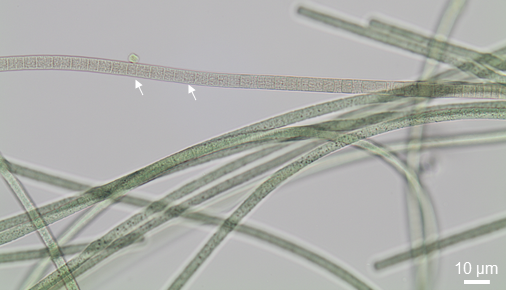


Supplementary Figure S2. Light micrographs of *Limnofasciculus delicatus* mat from Shenandoah River mixed with *Microcoleus* filament (arrows). Scale bar = 10 µm
